# Development and assessment of a pediatric emergency medicine simulation and skills rotation: meeting the demands of a large pediatric clerkship

**DOI:** 10.3402/meo.v20.29618

**Published:** 2015-11-30

**Authors:** Elaine K. Fielder, Daniel S. Lemke, Cara B. Doughty, Deborah C. Hsu, Amy B. Middleman

**Affiliations:** 1 Section of Emergency Medicine, Department of Pediatrics, Baylor College of Medicine, Texas Children's Hospital, Houston, TX, USA; 2 Section of Adolescent Medicine, Department of Pediatrics, University of Oklahoma Health Sciences Center, Oklahoma City, OK, USA

**Keywords:** pediatric clerkship, procedural training, simulation, overcrowding

## Abstract

**Objective:**

To implement a curriculum using simulation and skills training to augment a Pediatric Emergency Medicine (PEM) rotation within a pediatric clerkship.

**Background:**

PEM faculty are often challenged with a high learner to teacher ratio in a chaotic clinical setting. This challenge was heightened when our pediatric clerkship's traditional 1-week PEM rotation (consisting of 4 students completing four 8-hour ED shifts/week) expanded to 8 students every 2 weeks. We sought to meet this challenge by integrating simulation-based education into the rotation.

**Methods:**

Clerkship students from March to June 2012 completed our traditional rotation. Students between July and October 2012 completed the new PEM-SIM curriculum with 19 hours ED shifts/week and 16 hours/week of simulation/skills training. Pre/post-tests evaluated 1) medical management/procedural comfort (five-point Likert scale); and 2) PEM knowledge (15 multiple-choice questions).

**Results:**

One hundred and nine students completed the study (48 traditional, 61 PEM-SIM). Improvement in comfort was significantly higher for the PEM-SIM group than the traditional group for 6 of 8 (75%) medical management items (*p*<0.05) and 3 of 7 (43%) procedures, including fracture splinting, lumbar puncture, and abscess incision/drainage (*p*<0.05). PEM-SIM students had significantly more improvement in mean knowledge compared to the traditional group (*p*<0.001).

**Conclusions:**

We have successfully integrated 16 hours/week of faculty-facilitated simulation-based education into a PEM rotation within our clerkship. This curriculum is beneficial in clinical settings with high learner to teacher ratios and when patient care experiences alone are insufficient for all students to meet rotation objectives.

Medical students do not often have opportunities to care for critically ill pediatric patients or perform emergency procedures during their core pediatric clerkship and are often forced to delay these live patient encounters until the first year of residency (1). Pediatric Emergency Medicine (PEM) provides an ideal setting for students to learn these skills; however, a high learner to teacher ratio, variable patient care experiences, and the often chaotic emergency environment makes it difficult for students to obtain adequate faculty supervision and feedback (2). The degree to which PEM is incorporated into the pediatric clerkship varies widely between institutions, and a globally accepted medical student curriculum in PEM is not currently available.

Simulation-based education (SBE) includes any educational activity that utilizes simulation aides to replicate clinical scenarios. SBE has been reported to be superior to traditional educational methodologies and associated with improved patient-related outcomes compared with no simulation (3–5). The Liaison Committee on Medical Education expects simulation experiences in the curriculum (6); however, limited faculty time and financial resources are common hurdles clerkship directors face when incorporating SBE into training (7, 8). A recent survey of Pediatric Clerkship Directors reported that although SBE is being used in 89% of programs, it is unclear which modalities are the most appropriate for meeting the goals of the clerkship (8).

Medical students at our institution complete a PEM rotation as part of the required 8-week pediatric clerkship. Our traditional 1-week PEM rotation consisted of patient care experiences during scheduled shifts in the pediatric Emergency Center (EC) at Texas Children's Hospital in Houston, Texas. To support the expansion of the PEM rotation from 4 students for 1 week to 8 students for 2 weeks, we developed and implemented a simulation and skills-based component that could accommodate the increased number of learners and provide a consistent educational experience. Modification of our traditional PEM rotation provided an opportunity to introduce interactive teaching methods through a SIM and procedural skills curriculum.

This article describes the development and integration of a 2-week PEM-SIM curriculum within an 8-week core pediatric clerkship. We sought to determine if PEM-SIM is associated with greater increases in students’ knowledge and comfort with medical management/procedures than our traditional shift-intensive curriculum.

## Methods

All medical students assigned to the pediatric clerkship between March 2012 and October 2012 were enrolled in the study. Institutional review board approval was obtained prior to learner enrollment.

Students in the clerkship from March 2012 through June 2012 completed our traditional PEM curriculum, consisting of 32 hours/week (four 8-hour shifts) of clinical shifts in the EC with bedside teaching from pediatric residents, PEM fellows, and attending physicians. Didactic sessions (3 hours/week) were led by PEM fellows.

**Fig. 1:**
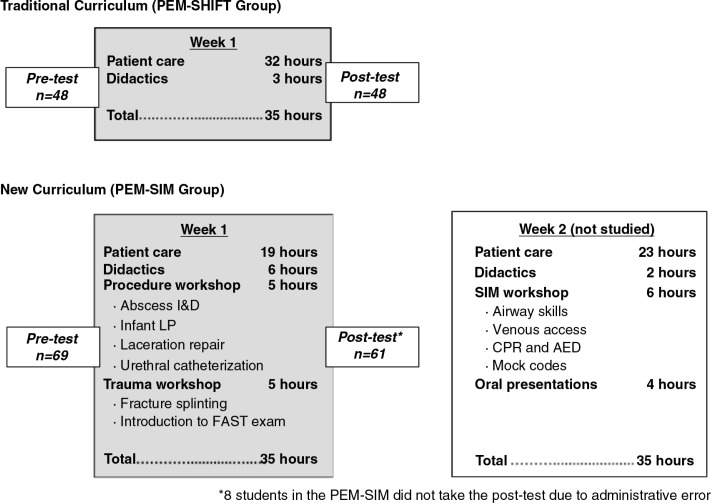
Study design and curriculum outline.

Students enrolled from July 2012 through October 2012 completed the new PEM-SIM curriculum that included 19 hours/week (one 7-hour weekday shift and one 12-hour weekend shift) of EC clinical shifts and 16 hours/week of procedural skills and simulation workshops. An overview of the PEM-SIM curriculum is described below and depicted in Fig. 1.

An assessment tool was derived from established rotation objectives and reviewed for accuracy and completeness by a group of medical educators and PEM experts. Students completed the assessment tool on the first day (pre-test) and after 1 week of the PEM rotation (post-test). All students completed 35 hours of training per week and were assessed after the first week to control for the difference in rotation length between study groups (Fig. 1).

The assessment tool included:

1. Comfort with 7 procedures and 8 medical management items measured with a Likert scale from 1 (very uncomfortable) to 5 (very comfortable). See Table 1.
2. PEM knowledge using 15 multiple-choice items recoded to dichotomous variables (correct/incorrect answers). Failure to respond to an item was scored as incorrect. See Table 2.

**Table 1:** Comfort with pediatric emergency procedures and medical management

	Traditional curriculum *n*=48			PEM-SIM curriculum *n*=61			
			
	Pre-test	Post-test	Δ	Pre-test	Post-test	Δ	Δ difference
Procedures							
Splinting a fracture^a^	1.69	2.94	+1.25	1.8	4.07	+2.27	+1.02*
Infant lumbar puncture^a^	1.81	2.40	+0.59	1.92	3.31	+1.39	+0.8*
Abscess I&D^a^	2.46	3.08	+0.62	2.79	3.98	+1.19	+0.57*
Laceration repair	2.42	2.94	+0.52	3.1	3.89	+0.79	+0.27
Endotracheal intubation	1.56	1.85	+0.29	1.89	2.28	+0.39	+0.1
PIV insertion	2.19	2.69	+0.50	2.59	3.2	+0.61	+0.11
Transurethral cath	2.67	3.04	+0.37	3.38	3.82	+0.44	+0.07
Medical management							
Closed head injury^a^	2.19	2.98	+0.79	2.00	3.57	+1.57	+0.78*
Neonatal fever^a^	3.13	3.65	+0.52	2.74	4.02	+1.28	+0.76*
Altered mental status	2.08	2.77	+0.69	1.90	3.30	+1.40	+0.71*
Vaso-occlusive crisis	2.25	2.69	+0.44	2.34	3.46	+1.12	+0.68*
Septic shock	2.13	2.67	+0.54	1.97	3.05	+1.08	+0.54*
Acute abdominal pain	2.94	3.71	+0.77	2.61	3.82	+1.21	+0.44*
Bronchiolitis	3.06	3.67	+0.61	2.92	3.75	+0.83	+0.22
Status asthmaticus	2.77	3.46	+0.69	2.57	3.31	+0.74	+0.1

Generalized linear model. Mean comfort score on a 0–5 point scale.

a Items taught in week 1 of the new PEM-SIM curriculum.

* *p*<0.05.

**Table 2:** Mean scores for knowledge assessment

	Traditional (*n*=48)			PEM-SIM (*n*=61)			
			
	Pre-test	Post-test	Δ	Pre-test	Post-test	Δ	p*
Mean score (SD)^a^	7.6 (2.1)	8.6 (2.9)	+1	7.5 (2.1)	10.4 (1.8)	+2.9	<0.001

* Mann–Whitney U.

a Score=number correct out of 15 questions.

## PEM-SIM curriculum (Figure 1)

Interactive workshops occurred twice weekly. Week 1 focused on fundamental PEM knowledge and procedural skills. Didactics included a PEM orientation, procedural overview (including informed consent and sterile technique), orthopedic injuries, head trauma, and an introduction to Focused Assessment with Sonography for Trauma (FAST). Students used task trainers to perform infant lumbar puncture (LP) and urethral catheterization. We used oranges as abscess models for incision and drainage (I&D). Students practiced laceration repair using suture blocks and slightly dampened sponges. Students performed fracture splinting and FAST exams on EC patients or volunteers from the student group. Week 2 covered advanced PEM topics including respiratory distress/failure, shock, and cardiopulmonary arrest. Full-body low-fidelity mannequins were used to practice pediatric airway skills, cardiopulmonary resuscitation, and intraosseous needle insertion. Partial task trainers were used to teach intravenous (IV) placement. Students practiced team-based communication and their recently learned emergency skills by participating in a variety of pediatric code simulations during the final workshop of the rotation.

Curriculum content was developed by a committee of PEM faculty, simulation experts, and the clerkship director, based on established clerkship objectives and published adult EM curricula (3, 9, 10). All workshops were led by PEM faculty members. Direct observation and formative feedback was provided to students throughout all sessions. To facilitate uniform instruction, teaching faculty were required to complete a 5-hour ‘Train the Trainer’ workshop that included a curriculum overview, feedback skills, and guidelines for task trainer use.

### Cost

The initial implementation costs of the PEM-SIM curriculum are outlined in Table 3. Implementation costs were significantly higher compared to subsequent years, as task trainers, mannequins, and durable supplies were reused for multiple sessions. Costs do not include faculty or fellow compensation.

**Table 3:** Approximate startup costs for the PEM-SIM curriculum

AED trainer	$407.00
Pediatric PIV task trainer set	$400.00
Urinary catheter male/female trainer set	$2,907.00
Infant lumbar puncture task trainer	$506.00
ALS infant mannequin	$2,343.60
Adult intubation task trainer	$1,895.00
3.0 nylon sutures	$36.98/box (12)
20 gauge PIV catheters	$123.40/case (200)
Urinary catheters 12 Fr 16"	$7.60/box (10)
Abscess incision/drainage set	$83.48/box (20)
Suture removal kit (scissors/forceps)	$93.00/box (50)
Pediatric AMBU bag/mask	$11.86
3 inch compression bandages	$9.75/box (10)
3 inch Webril	$16.21/bag (20)
3 inch×9 ft plaster rolls	$10.65/box (12)
Basic kitchen sponges (laceration models)	$3.99 (12)
Ripe oranges (abscess models)	$5.99/bag (6)
Approximate startup costs	$8,861.51

## Data analysis

Differences in pre-test comfort between groups were determined using Mann–Whitney U test. Differences in pre- and post-tests were analyzed using the generalized linear model. Associations between traditional versus PEM-SIM demographics (gender, age, and number of previous core rotations) were measured using chi-square analysis. Overall knowledge score was determined by recording the number of questions correct out of 15; mean scores are reported for ease of interpretation, and differences between groups were determined using Mann–Whitney U test. Statistical analyses were performed using SPSS.

## Results

One hundred and seventeen students completed the pre-test; 8 students in the PEM-SIM group were not administered the post-test in error and were omitted from analyses. One hundred and nine students completed pre- and post-tests: 48 in the traditional group and 61 in the PEM-SIM group (Fig. 1). There were no statistical differences between groups regarding gender, age, and number of previously completed core rotations.

### Comfort level (Table 1)

#### Procedural skills

Pre-test comfort was significantly higher in the PEM-SIM group for laceration repair and urethral catheterization (*p*=.010; *p*=.013, respectively). Baseline comfort was similar between groups for all other procedures. Comfort level improved significantly in the PEM-SIM group for fracture splinting, abscess I&D, and infant LP.

#### Medical management

Pre-test comfort was similar between groups for all medical management items. Students in the PEM-SIM group experienced greater increases in comfort with medical management topics with the exception of bronchiolitis and status asthmaticus.

### Knowledge (Table 2)

Baseline knowledge scores were similar in both groups. Both groups showed improved knowledge (as measured by the total number of correct answers out of 15 questions) after 1 week of the PEM rotation. The difference between pre- and post-test scores was significantly greater for the PEM-SIM group than for the traditional group.

## Discussion

Medical students who completed 1 week of the new PEM-SIM curriculum reported significantly greater improvements in comfort with most procedures and medical management and demonstrated greater improvements in mean knowledge scores than students who completed the traditional curriculum. Our study suggests that a PEM curriculum augmented with procedural skills and simulation teaching techniques may be superior to a traditional shift-intensive, patient-centered model.

Not all items showed significant improvement in the PEM-SIM group. Because week 2 of training was not assessed in this study, we were unable to evaluate the educational benefit of the complete PEM-SIM curriculum. For example, comfort with endotracheal intubation and PIV insertion did not improve significantly; however, these procedures were not taught until week 2. Laceration repair and urethral catheterization comfort levels also did not significantly improve; however, high pre-test comfort was already reported by the PEM-SIM group for these procedures.

Comfort levels improved for all medical management items formally taught in week 1 of the PEM-SIM curriculum, yet improvement was also seen with some medical management items not included in a didactic or workshop setting in week 1. Whether this finding is associated with meaningful patient interactions, self-study, or other learning modalities is unknown.

There were limitations to our study. Although both groups received 35 hours of instruction per week, our study design compares an intensive educational intervention that includes 6 hours of faculty-led didactics to a traditional shift model with 3 hours of unstructured didactic content. This difference may have contributed to the higher knowledge scores seen in the PEM-SIM group. We also cannot distinguish if student comfort improved from incorporating focused didactics or from the procedural training itself. Novices are historically poor at self-assessment when compared to competency measurements; therefore, self-reported comfort is not as strong an outcome as assessing competency using standardized checklists. In addition, students in the traditional and PEM-SIM groups were enrolled based on the predetermined dates of their clerkship assignments and were not randomized.

Despite these limitations, our study supports alternative educational modalities to offset decreased direct patient care hours. We successfully incorporated a 2-week PEM procedural skills and simulation-based curriculum within our 8-week pediatric clerkship and have sustained this model since its inauguration in July 2012. While not generalizable to all clerkships or their components, this curricular model can be adapted in settings with high learner to faculty ratios, limited faculty supervision, inadequate variety of patients, or disease rarity.
